# MAPK p38 regulates inflammatory gene expression via tristetraprolin: Doing good by stealth

**DOI:** 10.1016/j.biocel.2017.11.003

**Published:** 2018-01

**Authors:** J.D. O’Neil, A.J. Ammit, A.R. Clark

**Affiliations:** a Institute of Inflammation and Ageing, University of Birmingham, Birmingham B15 2WB, United Kingdom; b Woolcock Emphysema Centre, Woolcock Institute of Medical Research, University of Sydney, New South Wales, Australia; c School of Life Sciences, Faculty of Science, University of Technology, Sydney, New South Wales, Australia

**Keywords:** MAPK p38, Tristetraprolin, Post-transcriptional regulation, Inflammation, Adenosine/uridine-rich element

## Abstract

Tristetraprolin (TTP) is an RNA-destabilizing protein that exerts profound anti-inflammatory effects by inhibiting the expression of tumour necrosis factor and many other inflammatory mediators. The mitogen-activated protein kinase (MAPK) p38 signaling pathway controls the strength and duration of inflammatory responses by regulating both the expression and function of TTP. The kinase MK2 (MAPK activated kinase 2) is activated by MAPK p38, and in turn phosphorylates TTP at two critical serine residues. One consequence of these phosphorylations is the protection of TTP from proteasome-mediated degradation. Another consequence is the loss of mRNA destabilizing activity. The control of TTP expression and function by the MAPK p38 pathway provides an elegant mechanism for coupling the on and off phases of inflammatory responses, and dictating the precise kinetics of expression of individual inflammatory mediators.

## Highlights

• TTP negatively regulates expression of large numbers of inflammatory mediators.
• A TTP knock-out mouse suffers chronic and severe inflammatory pathology.
• TTP is multiply phosphorylated. Phosphorylation of serines 52 and 178 is mediated by the MAPK p38-activated kinase MK2.
• Phosphorylation of TTP at serines 52 and 178 causes its inactivation, and is permissive for expression of TTP-regulated inflammatory mediators.
• Inflammation is reduced by genetically or pharmaceutically interfering with the phosphorylation of serines 52 and 178.

## 1 Introduction

The mitogen-activated protein kinase (MAPK) p38 signaling pathway and the first generation of selective MAPK p38 inhibitors were both discovered in the mid 1990s (Arthur and Ley, 2013). Mammalian genomes encode four distinct MAPK p38 isoforms known as α, β, γ and δ. MAPK p38α and β are commonly activated by stressful or pro-inflammatory stimuli, and selectively inhibited by the drugs first identified at SmithKline Beecham. These compounds and selective inhibitors generated by other pharmaceutical companies reduced the expression of many inflammatory mediators, exerted therapeutic effects in several experimental models of inflammatory pathology, and ultimately underwent clinical trials in various chronic inflammatory diseases. Although few of these trials were reported in full, the outcomes appear to have been uniformly and surprisingly negative, leading one commentator to conclude that “The era of optimism surrounding the use of MAPK p38 inhibition … is over” (Genovese, 2009). It remains open to question whether the MAPK p38 pathway could still be therapeutically targeted in inflammatory disease, for example by attacking different points in the signaling cascade, by inhibiting MAPK p38 itself in a different manner, or by selecting different inflammatory pathologies for treatment. Judgement of this issue requires a more complete understanding of how MAPK p38 regulates expression of inflammatory mediators, and why such promising pre-clinical data failed to translate into clinical efficacy.

## 2 Post-transcriptional regulation of inflammatory responses

Although it is scarcely mentioned in some reviews, MAPK p38 regulates inflammatory responses largely at a post-transcriptional level. Post-transcriptional regulation in the innate immune system has been reviewed extensively (Carpenter et al., 2014, Tiedje et al., 2014). Many of the factors induced by infection or injury have powerful, pleiotropic effects and can cause severe damage if their expression is unchecked. Therefore rapid on-and-off switching of gene expression is fundamental to the innate immune system. This type of regulation requires rapid mRNA turnover, otherwise dynamic changes of transcription rate will be negated by the long-lasting intermediates. Hence short half-lives are highly characteristic of many inflammatory mediator mRNAs. In the majority of cases deadenylation (the removal of the protective 3′ poly-(A) tail) is the rate limiting step in mRNA degradation. Deadenylation is regulated by sequences located within mRNA 3′ untranslated regions (UTRs), which act as cognate sites for sequence-specific RNA binding proteins. The best characterized of these regulatory sites are the adenosine/uridine-rich elements (AREs), which are common in the 3′ UTRs of inflammatory mRNAs, and often contain overlapping repeats of the motif AUUUA. Unsurprisingly, rates of mRNA degradation can be modulated in response to extracellular stimuli, providing additional mechanisms for the regulation of inflammatory responses. The physiological importance of post-transcriptional mechanisms to inflammatory and other immune responses has been illustrated by both genome-wide and gene-targeted approaches (Brooks and Blackshear, 2013, Kontoyiannis et al., 1999, Rabani et al., 2014, Ross et al., 2015).

## 3 Tristetraprolin as a mediator of post-transcriptional regulation by MAPK p38

Sustained MAPK p38 signaling is required for stabilization of many pro-inflammatory mRNAs in several different cell types (references in Tiedje et al., 2014). AREs in the 3′ UTRs of these transcripts are necessary and sufficient for post-transcriptional regulation by MAPK p38 (Dean et al., 2003, Kontoyiannis et al., 1999, Marchese et al., 2010, Tiedje et al., 2014, Tudor et al., 2009). Different experimental approaches indicated that the effects of MAPK p38 are mediated by the downstream kinase MK2 (MAPK-activated kinase 2) (Tiedje et al., 2014). Finally, MAPK p38 delayed the decay of target mRNAs by inhibiting their deadenylation (Dean et al., 2003). These observations led to the hypothesis that an ARE-binding protein promotes the deadenylation and subsequent degradation of target mRNAs, and this protein can be inactivated by MK2 to promote mRNA stabilization.

**Fig. 1:**
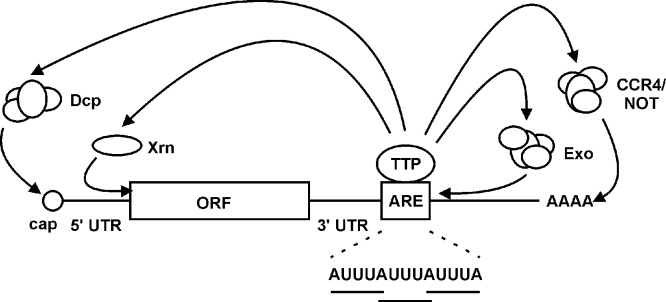
TTP interacts with multiple mRNA-processing factors. TTP interacts in a sequence-specific manner with an adenosine/uridine-rich element (ARE) in the 3′ untranslated region (UTR) of a target mRNA. A typical ARE sequence is shown below the schematic. TTP can then interact with the CCR4/NOT complex to promote shortening of the poly-(A) tail; with the exosome (Exo) to promote degradation of the mRNA body in the 3′ to 5′ direction; with the decapping complex (Dcp) to promote removal of the protective 7-methylguanosine cap at the 5′ end of the mRNA; with exoribonuclease 1 (Xrn) to promote degradation of the mRNA body in the 5′ to 3′ direction. ORF, open reading frame.

The clearest and most consistent evidence points to tristetraprolin (TTP) as a mediator of post-transcriptional effects of the MAPK p38 pathway (Clark and Dean, 2016). Other putative mediators are discussed elsewhere (Tiedje et al., 2014). TTP, the product of the *Zfp36* gene in mouse and the *ZFP36* gene in man, is a principally cytoplasmic protein of approximately 36 kD, and interacts with RNA via a central domain containing two C3H-type zinc fingers (ie. zinc is coordinated by three cysteine and one histidine residues) (Brooks and Blackshear, 2013). Its consensus core binding motif, UAUUUAU, is found in the 3′ UTRs of MAPK p38-sensitive mRNAs. The *Zfp36* knockout mouse has a severe, spontaneous inflammatory phenotype in which the dominant pathological features are driven by increased stability of tumour necrosis factor (*Tnf*) mRNA and dysregulated expression of TNF protein. However, growing numbers of genes are also found to be post-transcriptionally dysregulated in *Zfp36-/-* cells, contributing to different aspects of the phenotype (Brooks and Blackshear, 2013). This reflects the fact that TTP binds to hundreds of mRNA targets, many of which are involved in cellular responses to stressful and pro-inflammatory stimuli (Sedlyarov et al., 2016, Tiedje et al., 2016). Once bound to target transcripts, TTP promotes their degradation by recruiting various nucleolytic protein complexes (Fig. 1). Amongst these, the most important is likely to be the CCR4-NOT (carbon catabolite repression 4 − negative on TATA-less) complex, which mediates mRNA deadenylation. Importantly, MAPK p38 inhibitors failed to destabilize several inflammatory mRNAs or to inhibit the expression of the corresponding proteins in *Zfp36-/-* macrophages (Carballo et al., 2001, Tudor et al., 2009).

## 4 Mechanisms of regulation of TTP by the MAPK p38 pathway

TTP is expressed at very low levels in resting macrophages, and as it is synthesized in response to LPS (lipopolysaccharide) it becomes extensively phosphorylated (Mahtani et al., 2001). More than 30 sites of phosphorylation of TTP have been documented, but only a few studied in detail (Clark and Dean, 2016). *In vitro* TTP is efficiently phosphorylated by MK2 at serines 52, 178 and 316 (mouse coordinates) (Chrestensen et al., 2004, Mahtani et al., 2001, Stoecklin et al., 2004). The phosphorylation of S52 and S178 promotes binding of 14-3-3 proteins, abundant low molecular-weight adaptors that specifically recognize phosphorylated client proteins and help to bring about phosphorylation-mediated changes of localization and function. As a consequence of these phosphorylations, the recruitment of the CCR4-NOT complex is impaired, the localization of TTP to cytoplasmic sites of mRNA turnover is altered, and target mRNAs are stabilized (Clement et al., 2011, Marchese et al., 2010, Stoecklin et al., 2004). S316 lies within a highly conserved C-terminal CCR4-NOT recruitment domain. Its phosphorylation is also thought to impair recruitment of the deadenylase complex, although likely by a different mechanism (Fabian et al., 2013).

**Fig. 2:**
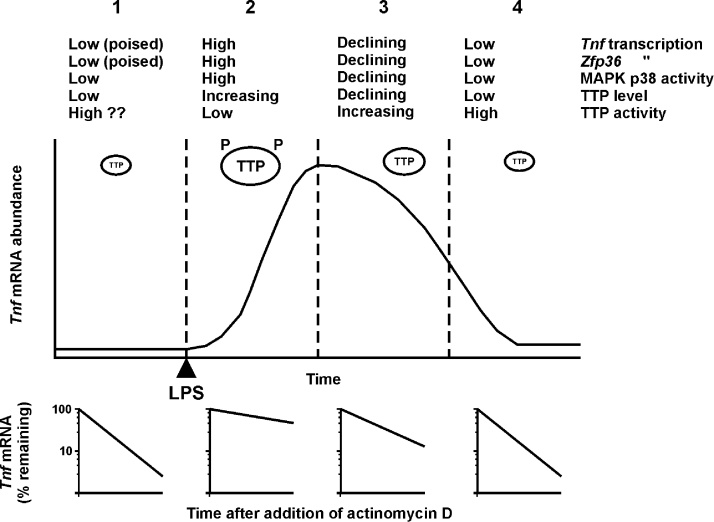
Phosphorylation-regulated function of TTP during the evolution of an inflammatory response. *Tnf* is used as an exemplar TTP-regulated transcript here. 1. In the resting state TTP is expressed at very low levels. Any TTP present is likely to be predominantly unphosphorylated at S52 and S178, and may be restricted to the nucleus (Brook et al., 2006). Nuclear functions of TTP are not clear. One possibility is that nuclear TTP may help to prevent leaky expression of pro-inflammatory mediators such as TNF, which often have constitutive RNA polymerase 2 recruitment at their promoters. 2. In respose to an inflammatory challenge, expression of TTP is accompanied by its MK2-mediated phosphorylation at S52 and S178. These phosphorylations render TTP inactive, and target mRNAs are initially stable. 3. As MAPK p38 activity declines, the pool of inactive TTP is dephosphorylated by MK2 and becomes active. Target mRNAs are consequently destabilized, driving the off-phase of gene expression. The exact tipping point between on- and off-phases depends on the strength and duration of transcriptional activation and (possibly) the affinity of the particular mRNA for TTP (McGuire et al., 2016, Tang et al., 2017). 4. Ubiquitin-independent degradation of TTP protein facilitates a return to ground state.

TTP lacks defined structure outside its central zinc finger domain, and is targeted for degradation by the proteasome via an unfolded protein response (Ngoc et al., 2014). Phosphorylation of S52 and S178 prevents TTP protein degradation (Brook et al., 2006, Hitti et al., 2006, Ngoc et al., 2014), possibly via imposition of stable structure. Dephosphorylation and activation of TTP can be carried out by protein phosphatase 2A (PP2A), but this is accompanied by a decrease in TTP protein stability (Brook et al., 2006, Frasca et al., 2010, Mahtani et al., 2001, Rahman et al., 2016, Sun et al., 2007). As outlined in Fig. 2, the dynamic equilibrium between phosphorylated (inactive) and unphosphorylated (active) TTP is believed to contribute to the precise temporal orchestration of inflammatory gene expression by the MAPK p38 pathway (Clark and Dean, 2016, Kratochvill et al., 2011, Prabhala et al., 2015, Rabani et al., 2014, Sedlyarov et al., 2016). The coupling between TTP protein stability and mRNA destabilizing activity may also help to explain the loss of efficacy of MAPK p38 inhibitors in clinical trials, as explored in more detail elsewhere (Clark and Dean, 2016). Another interesting consequence is that the quantity of TTP protein in a cell or tissue may be inversely related to its mRNA destabilizing activity. This could explain the abundance of TTP found at sites of inflammation such as the rheumatoid synovium, where it is co-localized with active MAPK p38 (Ross et al., 2017).

## 5 A TTP knock-in mouse with an inflammation-resistant phenotype

To test the working model described above, we generated a knock-in mouse strain, in which serines 52 and 178 of endogenous TTP protein were substituted by non-phosphorylatable alanine residues (Ross et al., 2015). As predicted, TTP was expressed at very low levels in genetically modified (*Zfp36aa/aa*) primary macrophages, to the extent that it was almost undetectable in some experiments. However, it functioned very efficiently as an mRNA destabilizing factor, and strongly inhibited the expression of many inflammatory mediators (McGuire et al., 2016, O'Neil et al., 2017, Ross et al., 2015, Tang et al., 2017). *In vivo* the targeted mutagenesis of the *Zfp36* gene conferred strong protection in an experimental model of endotoxemia (Ross et al., 2015) and complete protection in an experimental model of rheumatoid arthritis (Ross et al., 2017). The hypo-inflammatory phenotype of *Zfp36aa/aa* mice was accompanied by striking decreases in the expression of many inflammatory mediators *in vivo*, for example an almost 200-fold lower serum level of IL-6 after injection of LPS (O'Neil et al., 2017, Ross et al., 2015). Nevertheless, the strong evolutionary conservation of the sites of MK2-mediated phosphorylation (Clark and Dean, 2016) implies that loss of MAPK p38-mediated control of TTP function confers a selective disadvantage. So far we have discovered only subtle alterations of adaptive immunity in *Zfp36aa/aa* mice (Ross et al., 2015), and no serious harmful consequences of the targeted mutation (O'Neil et al., 2017). In summary, the mutant form of TTP exerts profound anti-inflammatory effects despite its near undetectable levels of expression, a phenomenon we describe as, “Doing good by stealth”.

## 6 Dual specificity phosphatase 1 controls TTP function

Dual specificity phosphatase 1 (DUSP1) dephosphorylates and inactivates MAPK p38. Its expression is induced by pro-inflammatory stimuli, constituting a negative feedback loop to limit the duration of MAPK p38 signaling. Defective negative feedback control causes excessive inflammatory responses in *Dusp1-/-* mice and cells. The *Dusp1-/-* phenotype is largely explained by increased phosphorylation and inactivation of TTP, and consequent stabilization of TTP target transcripts (Smallie et al., 2015). The cross-talk between DUSP1 and TTP controls the expression of several important inflammatory mediators including TNF, IL-6 (interleukin 6), IL-1β, CXCL1 (chemokine CXC motif ligand 1), CXCL2, Cyclooxygenase 2 and Interferon β (McGuire et al., 2016, O'Neil et al., 2017, Smallie et al., 2015, Tang et al., 2017). An implication is that agonists which enhance or prolong the expression of DUSP1 could reduce expression of these genes and exert anti-inflammatory effects by modulating the equilibrium between phosphorylated and unphosphorylated TTP. Prostaglandin E_2_ (PGE_2_) exerts anti-inflammatory effects in this way (Rumzhum and Ammit, 2016). Whilst *Dusp1-/-* macrophages over-express TNF and *Zfp36aa/aa* macrophages under-express TNF, both are similarly insensitive to the anti-inflammatory effects of PGE_2_ (Tang et al., 2017). Other agonists that elevate intracellular cAMP and enhance DUSP1 expression may exert anti-inflammatory effects via the same mechanism. Glucocorticoids are well known to increase the expression of DUSP1, and their anti-inflammatory effects are also partly mediated by modulation of TTP function (Prabhala et al., 2016).

## 7 The critical role of PP2A

The dephosphorylation and activation of phosphorylated TTP is mediated by PP2A (Sun et al., 2007). Both under resting conditions and after stimulation of cells with TNF, PP2A restrains the expression of inflammatory genes, at least in part by controlling TTP function (Rahman et al., 2016, Rahman et al., 2015). It follows that targeted stimulation of PP2A should exert anti-inflammatory effects by enhancing TTP function. This prediction has been tested and proven correct both *in vitro* and *in vivo*, using chemically distinct PP2A-activating molecules (Rahman et al., 2016, Ross et al., 2017). In primary macrophages, anti-inflammatory effects of one PP2A agonist were dependent on the presence of intact phosphorylation sites S52 and S178, and an intact TTP binding site in the target mRNA (Ross et al., 2017). We conclude that PP2A agonists may have therapeutic utility in chronic inflammatory diseases, where aberrant activation of the MAPK p38 pathway leads to the accumulation of TTP in a phosphorylated and inactive form.

## 8 Conclusion

The intimate link between MAPK p38 and TTP provides an elegant system for fine-tuning inflammatory responses, in terms of both the strength of response to challenge and the precise timing of on- and off-switches. The physiological significance of this mechanism is well illustrated by the dramatic inflammation-resistant phenotype arising from substitution of just two amino acids of endogenous TTP (McGuire et al., 2016, O'Neil et al., 2017, Ross et al., 2017, Ross et al., 2015, Smallie et al., 2015, Tang et al., 2017). At least in principle, this mechanism of control of inflammatory responses appears to be tractable as a novel therapeutic target (Rahman et al., 2016, Ross et al., 2017).

We lack space for discussion of other interesting matters such as the relationship between the MAPK p38 dependent phosphorylations and other phosphorylations of TTP (Clark and Dean, 2016), or the relationship between the turnover of TTP protein and that of its mRNA targets (Brooks, 2010). Phosphorylation of TTP clearly influences translation as well as stability of target mRNAs, which is neglected here but discussed elsewhere (Tiedje et al., 2014, Clark and Dean, 2016). The issue of timing is also too complex to do justice to here. For example, sustained inhibition of MAPK p38 depletes cells of TTP protein (Mahtani et al., 2001) and fails to destabilize target mRNAs (Tudor et al., 2009), suggesting that the timing of p38-targeting drug delivery should be explored in more detail. DUSP1-mediated negative feedback results in oscillations of MAPK p38 activity, which are required for efficient expression of inflammatory mediators (Tomida et al., 2015). It is not known whether oscillations of MAPK p38 activity influence TTP activity. Finally, the timing of stimuli that enhance DUSP1 expression is likely to be crucial (Tang et al., 2017). *In vitro* assays are poor at capturing the complexities of asynchronous and conflicting cell stimulations that exist in the real world. More sophisticated systems are required to explore the links between MAPK p38 and TTP *in vivo*.
